# Serum Interleukin-18, Fetuin-A, Soluble Intercellular Adhesion Molecule-1, and Endothelin-1 in Ankylosing Spondylitis, Psoriatic Arthritis, and SAPHO Syndrome

**DOI:** 10.3390/ijms17081255

**Published:** 2016-08-03

**Authors:** Hanna Przepiera-Będzak, Katarzyna Fischer, Marek Brzosko

**Affiliations:** 1 Department of Rheumatology, Internal Medicine and Geriatrics, Pomeranian Medical University in Szczecin, Unii Lubelskiej 1, Szczecin 71-252, Poland; brzoskom@pum.edu.pl; 2 Independent Laboratory of Rheumatic Diagnostics, Pomeranian Medical University in Szczecin, Unii Lubelskiej 1, Szczecin 71-252, Poland; kasia.f11@op.pl

**Keywords:** interleukin 18, fetuin-A, soluble intercellular adhesion molecule-1, endothelin-1, ankylosing spondylitis, psoriatic arthritis, SAPHO

## Abstract

To examine serum interleukin 18 (IL-18), fetuin-A, soluble intercellular adhesion molecule-1 (sICAM-1), and endothelin-1 (ET-1) levels in ankylosing spondylitis (AS), psoriatic arthritis (PsA), and Synovitis Acne Pustulosis Hyperostosis Osteitis syndrome (SAPHO). We studied 81 AS, 76 PsA, and 34 SAPHO patients. We measured serum IL-18, fetuin-A, sICAM-1, ET-1, IL-6, IL-23, vascular endothelial growth factor (VEGF), and epidermal growth factor (EGF). IL-18 levels were higher in AS (*p* = 0.001), PsA (*p* = 0.0003), and SAPHO (*p* = 0.01) than in controls, and were positively correlated with CRP (*p* = 0.03), VEGF (*p* = 0.03), and total cholesterol (TC, *p* = 0.006) in AS and with IL-6 (*p* = 0.03) in PsA. Serum fetuin-A levels were lower in AS (*p* = 0.001) and PsA (*p* = 0.001) than in controls, and negatively correlated with C-reactive protein (CRP) in AS (*p* = 0.04) and SAPHO (*p* = 0.03). sICAM-1 positively correlated with CRP (*p* = 0.01), erythrocyte sedimentation rate (ESR, *p* = 0.01), and IL-6 (*p* = 0.008) in AS, and with IL-6 (*p* = 0.001) in SAPHO. Serum ET-1 levels were lower in AS (*p* = 0.0005) than in controls. ET-1 positively correlated with ESR (*p* = 0.04) and Disease Activity Score 28 (DAS28, *p* = 0.003) in PsA. In spondyloarthritis, markers of endothelial function correlated with disease activity and TC.

## 1. Introduction

There is evidence of an increased risk of atherosclerosis and cardiovascular disease (CVD) in inflammatory rheumatic diseases [1,2,3,4,5,6,7,8,9]. Ankylosing spondylitis (AS), psoriatic arthritis (PsA), and SAPHO syndrome (SAPHO) are seronegative spondyloarthropathies (SpA) [10,11,12].

Inflammation in the course of arthritis, as well as traditional risk factors of atherosclerosis together with cytokines and adhesion molecules, influence endothelial activation and dysfunction. Interleukin 18 (IL-18), fetuin-A, soluble intercellular adhesion molecule-1 (sICAM-1), and endothelin-1 (ET-1) are a group of cytokines and adhesion molecules which activate and deregulate endothelial function, and could increase the risk of atherosclerosis [13,14,15,16,17,18,19,20]. Serum interleukin 6 (IL-6) and interleukin 23 (IL-23) are considered to be associated with the inflammatory process in spondyloarthritis [21,22]. Additionally, IL-6 stimulates the hepatic production of fibrinogen and is also involved in endothelial activation [22]. Angiogenesis, stimulated by vascular endothelial growth factor (VEGF) and epidermal growth factor (EGF), also plays a role in the pathogenesis of SpA [23,24,25,26].

The aim of the study was to examine serum IL-18, fetuin-A, sICAM-1, and ET-1 levels as markers of endothelial function in correlation with disease activity and the lipid profile in AS, PsA, and SAPHO.

## 2. Results

The clinical, laboratory characteristics and treatment of the patients and controls are presented in Table 1.

**Figure 1:**
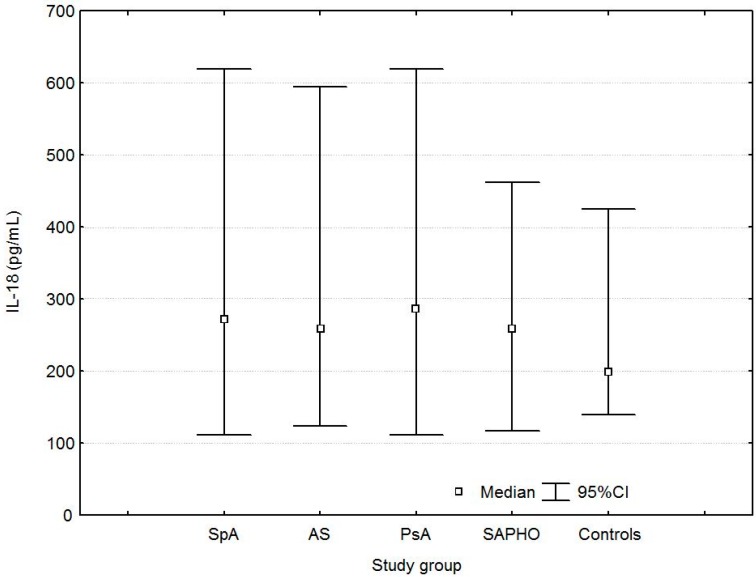
Serum concentrations of interleukin-18 (IL-18) in patients with spondyloarthritis (SpA), ankylosing spondylitis (AS), psoriatic arthritis (PsA), SAPHO syndrome (SAPHO), and controls.

Serum IL-18 levels were higher in SpA, AS, PsA, and SAPHO patients than in controls (Table 2, Figure 1).

**S**erum IL-18 levels were higher in male patients than in female patients with PsA (347.1 (269.8–429.6) vs. 269.6 (200.5–306.4) pg/mL, *p* = 0.003) and in controls (227.0 (206.9–273.1) vs. 190.0 (160.3–248.8) pg/mL, *p* = 0.05). No differences in serum IL-18 levels were found between the AS, PsA, and SAPHO groups (*p* > 0.05).

SpA patients had an increased risk of increased serum IL-18 (Table 3) compared to controls.

**Figure 2:**
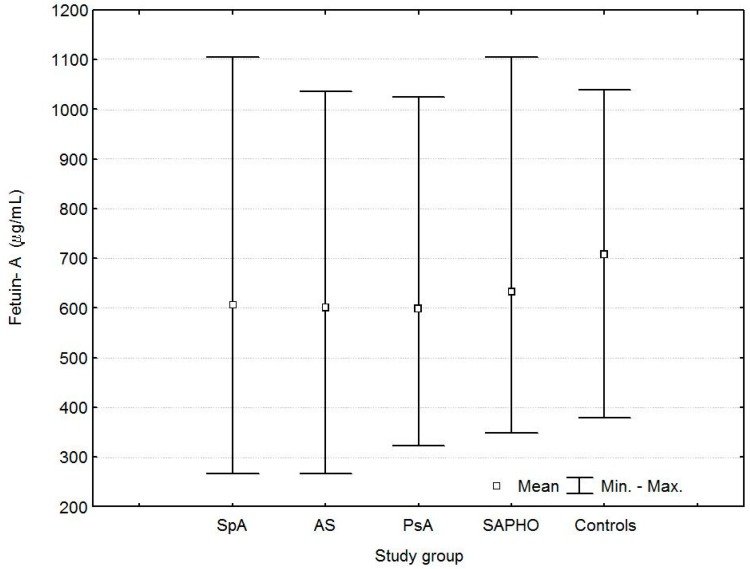
Serum concentrations of fetuin-A in patients with spondyloarthritis (SpA), ankylosing spondylitis (AS), psoriatic arthritis (PsA), SAPHO syndrome (SAPHO), and controls.

Serum fetuin-A levels were lower in SpA, AS, and PsA patients than in controls. (Table 2, Figure 2).

In AS patients, serum fetuin-A levels were lower in males than in females (577.2 ± 138.1 vs. 673.7 ± 156.1 µg/mL, *p* = 0.009). No differences were found between female and male patients in terms of serum fetuin-A levels in the PsA, SAPHO, and control groups (all *p* > 0.05). No differences were observed in serum fetuin-A levels between the AS, PsA, and SAPHO groups (*p* > 0.05).

SpA patients had an increased risk of decreased serum fetuin-A compared to controls (Table 3).

No differences were found between SpA patients and controls in terms of sICAM-1 levels (Table 2). No differences were found between females and males in terms of sICAM-1 in SpA patients and in controls (all *p* > 0.05). No differences were found in serum sICAM-1 levels between the AS, PsA, and SAPHO groups (all *p* > 0.05). 

SpA patients, compared to controls, had an increased risk of increased serum sICAM-1 levels (Table 3).

**Figure 3:**
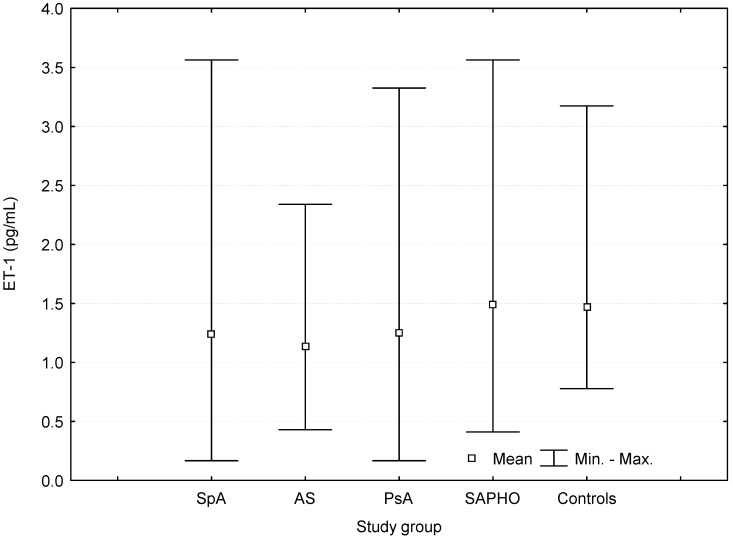
Serum concentrations of endothelin-1 (ET-1) in patients with spondyloarthritis (SpA), ankylosing spondylitis (AS), psoriatic arthritis (PsA), SAPHO syndrome (SAPHO), and controls.

Serum ET-1 levels were lower in SpA and AS patients than in controls. (Table 2, Figure 3).

Serum ET-1 levels were lower in AS than in SAPHO patients (*p* = 0.0003), and in PsA than in SAPHO patients (*p* = 0.05, Table 2). No differences were found between female and male patients in terms of serum ET-1 levels in SpA patients and in controls (all *p* > 0.05). 

SpA patients had an increased risk of decreased serum ET-1 levels compared to controls (Table 3).

There was a positive correlation between serum IL-18 and sICAM-1 in SpA (*r* = 0.3; *p* = 0.00001), AS (*r* = 0.22; *p* = 0.04), and PsA (*r* = 0.43; *p* = 0.0001) patients. No correlations were found between IL-18 and fetuin-A or ET-1.

In AS patients, sICAM-1 was positively correlated with ET-1. No correlations were found between sICAM-1 and fetuin-A in SpA.

### 2.1. Markers of Endothelial Function and Disease Activity in SpA

There was a positive correlation between serum IL-18 and CRP (*r* = 0.24; *p* = 0.03) in AS patients. There was a positive correlation between serum IL-18 with IL-6 (*r* = 0.27; *p* = 0.03) and with BASDAI (*r* = 0.24; *p* = 0.03) in PsA patients. No correlations were found between serum IL-18 and disease activity in the SAPHO group.

The assessment of the age–sex adjusted *p*-value by ANOVA confirmed the positive correlation between serum IL-18 and VEGF in SpA (*r* = 0.28; *p* = 0.004) and AS (*r* = 0.33; *p* = 0.03) patients.

No correlations were found between serum IL-18 and VAS, ASDAS-ESR, DAS28, IL-23, EGF, or ERS (all *p* > 0.05) in SpA patients.

There was a negative correlation between serum fetuin-A and CRP in AS (*r* = −0.23; *p* = 0.04) and SAPHO (*r* = −0.38; *p* = 0.03) patients. SAPHO patients with CRP ≥ 5 mg/L had lower fetuin-A compared to those with CRP < 5 mg/L (547.1 ± 143.3 vs. 690.0 ± 188.3 pg/mL, *p* = 0.02). There was a positive correlation between fetuin-A and VEGF in SpA (*r* = 0.32; *p* = 0.001) and AS (*r* = 0.4; *p* = 0.007) patients.

The assessment of the age–sex adjusted *p*-value by ANOVA for SpA patients with CRP ≥ 5 mg/L confirmed the positive correlation between serum fetuin-A and CRP (*r* = 0.22; *p* = 0.002). Additionally, in SpA patients with VAS > 40, there was a positive correlation between serum fetuin-A and VAS (*r* = 0.21; *p* = 0.004). There was positive correlation between serum fetuin-A and IL-6 in SpA (*r* = 0.21; *p* = 0.01) and in AS (*r* = 0.29; *p* = 0.02) patients. There was a positive correlation between serum fetuin-A and IL-23 in SAPHO patients (*r* = 0.5; *p* = 0.01).

No correlations were found between serum fetuin-A and BASDAI, ASDAS-ESR, DAS28, EGF, or ERS (all *p* > 0.05) in SpA patients.

There was a positive correlation between serum sICAM-1 and IL-6 in AS (*r* = 0.34; *p* = 0.008) and SAPHO (*r* = 0.5; *p* = 0.001) patients. In AS patients, there was a positive correlation between serum sICAM-1 and CRP (*r* = 0.28; *p =* 0.01), ESR (*r* = 0.29; *p =* 0.01), and age (*r* = 0.2; *p =* 0.006). AS patients with CRP ≥ 5 mg/L, compared with AS patients with CRP < 5 mg/L, had higher serum sICAM-1 (238.9 ± 62.7 vs. 208.2 ± 40.4 ng/mL, *p* = 0.02). No correlations were found between sICAM-1 and VAS, BASDAI, ASDAS-ESR, DAS28, IL-23, VEGF, or EGF (all *p* > 0.05) in SpA patients.

In PsA patients, serum ET-1 was positively correlated with ESR (*r* = 0.24; *p* = 0.04) and DAS28 (*r* = 0.7; *p* = 0.003). SpA patients with ESR ≥ 10 had higher levels of ET-1 compared to those with ESR < 10 (1.32 ± 0.6 vs. 1.11 ± 0.4 pg/mL, *p* = 0.02).

There was a negative correlation between ET-1 and EGF in AS (*r* = −0.31; *p =* 0.04) and in SAPHO (*r* = −0.83; *p =* 0.0002) patients. 

The assessment of the age–sex adjusted *p*-value by ANOVA confirmed the positive correlation between serum ET-1 and IL-6 in SpA (*r* = 0.25; *p* = 0.001) and AS (*r* = 0.27; *p* = 0.03) patients.

No correlations were found between ET-1 and VAS, BASDAI, ASDAS-ESR, IL-23, VEGF, or CRP (all *p* > 0.05) in SpA patients.

SpA patients with a BASDAI score > 4 and those with a score ≤ 4 did not differ in terms of their levels of IL-18, fetuin-A, sICAM-1, and ET-1 (all *p* > 0.05)

SpA patients with a VAS score > 40 and those with a score ≤ 40 did not differ in terms of their levels of IL-18, sICAM-1, and ET-1 (all *p* > 0.05). However, SpA patients with a VAS score > 40 had lower levels of fetuin-A (584.9 ± 144.4 vs. 664.9 ± 171.9 pg/mL, *p* = 0.005) compared to those with a VAS score ≤ 40. SAPHO patients with a VAS score > 40 had lower levels of fetuin-A (573.2 ± 124.0 vs. 800.8 ± 214.5 pg/mL, *p* = 0.005) compared to those with a VAS score ≤ 40.

### 2.2. Markers of Endothelial Function and HLA-B27 in SpA

AS patients positive for HLA-B27 had higher serum sICAM-1 compared to those negative for HLA-B27 (228.7 ± 70.9 vs. 158.1 ± 34.3 ng/mL, *p* = 0.02) and ET-1 (1.07 ± 0.31 vs. 0.67 ± 0.22 pg/mL, *p* = 0.03). 

PsA patients positive for HLA-B27 had higher serum sICAM-1 compared to those negative for HLA-B27 (281.7 ± 74.8 vs. 212.4 ± 46.3 ng/mL, *p* = 0.04).

No significant associations between HLA-B27 positivity and serum IL-18 and fetuin-A levels were found.

### 2.3. Markers of Endothelial Function and BMI and WHR in SpA

SpA patients had a higher mean BMI than control subjects (*p* = 0.006). No differences in BMI were observed between AS, PsA, and SAPHO patients (*p* > 0.05, Table 1).

There was a positive correlation between serum IL-18 and BMI in AS (*r* = 0.28; *p* = 0.02) and SAPHO (*r* = 0.53; *p* = 0.01) patients. AS patients with BMI > 25.0 had higher IL-18 levels compared to those with BMI ≤ 25.0 (271.9 (228.7–454.5) vs. 245.8 (187.6–323.5) pg/mL, *p* = 0.04). SAPHO patients with BMI > 25.0 had higher IL-18 levels compared to those with BMI ≤ 25.0 (295.29 (210.2–428.4) vs. 221.4 (155.5–240.8) pg/mL, *p* = 0.04). 

There was a positive correlation between IL-18 and WHR in AS patients (*r* = 0.26; *p* = 0.04). AS patients with an increased WHR (for female ≥ 0.8, for male ≥ 1.0) had higher IL-18 levels compared to those with a normal WHR (402.7 (267.8–486.1) vs. 245.8 (205.3–357.3) pg/mL, *p* = 0.02). 

There was a positive correlation between sICAM-1 and BMI in AS patients (*r* = 0.21; *p* = 0.01). AS patients with BMI > 30 had higher sICAM-1 compared to AS patients with BMI < 25 (256.5 ± 41.4 vs. 219.8 ng/mL, *p* = 0.01). AS patients with BMI > 25 had higher ET-1 compared to those with BMI ≤ 25 (1.22 ± 0.35 vs. 1.04 ± 0.44 pg/mL, *p* = 0.04). 

No correlations were found between serum fetuin-A or ET-1 with BMI or WHR in SpA patients (all *p* > 0.05).

### 2.4. Markers of Endothelial Function and Lipid Profile in SpA

In AS patients, TC levels were lower than in controls (*p* = 0.001). In AS patients, LDL-C levels were lower than in controls (*p* = 0.02, Table 1).

There was a positive correlation between serum TC and IL-18 (*r* = 0.36; *p* = 0.006) and fetuin-A (*r* = 0.38; *p* = 0.004) in AS patients. Additionally, in AS patients, there was a positive correlation between fetuin-A and LDL-C (*r* = 0.34; *p* = 0.01) and TG (*r* = 0.38; *p* = 0.004). 

No correlations were found between the lipid profile and sICAM-1 or ET-1 in SpA patients (all *p* > 0.05).

### 2.5. Markers of Endothelial Function, Treatment, and Comorbidities in SpA

The results of the univariate and multivariate logistic regression analysis and step-wise analysis of serum IL-18, fetuin-A, sICAM-1, and ET-1 in terms of treatment with NSAIDs, NSAIDs with sulfasalazine, and NSAIDs with methotrexate showed no differences between different treatment groups in SpA patients (all *p* > 0.05).

AS patients with AAU had higher levels of IL-18 compared to those without AAU (323.5 (228.7–454.5) vs. 251.9 (201.8–324.7) pg/mL, *p* = 0.03).

The results of the univariate and multivariable logistic regression analysis and step-wise analysis of serum IL-18, fetuin-A, sICAM-1, and ET-1 levels in SpA patients who were smokers and non-smokers and with and without comorbidities such as IHD, hypertension, and diabetes showed no differences (all *p* > 0.05).

## 3. Discussion

Patients with chronic inflammatory arthritis such as AS, PsA, and SAPHO are to prone to the early development and acceleration of atherosclerosis [1,2,3,4,5,6,7,8,9]. The pathogenesis of atherosclerosis is connected to dyslipidemia and markers of inflammation (e.g., CRP and IL-6), as well as other cytokines and adhesion molecules which affect endothelial cell activation [3,4,15]. It has been shown that in patients with AS, a high serum IL-6 level is associated with a higher risk of atherosclerosis [2,22]. 

Sparse data are available regarding serum IL-18, fetuin-A, sICAM-1, and ET-1 in SpA patients, as these markers are mainly considered in AS [27,28,29,30,31,32,33,34,35].

Expression of IL-18 has been found in atherosclerotic plaques, localized mainly in plaque macrophages [13]. This cytokine is associated with atherosclerosis, and is a strong predictor of cardiovascular death in stable and unstable angina [14]. In our study, serum IL-18 was higher in SpA patients than in control subjects; additionally, SpA patients had an increased risk of increased serum IL-18. The same was found by Sari et al. [30] in AS and by Hurdado-Nedelec et al. [29] in SAPHO and PsA. Rooney et al. [28] observed increased synovial tissue IL-18 in PsA, reactive arthritis, and seronegative patients, before treatment was started. These authors found that after treatment, tissue IL-18 expression was changed, and they concluded that IL-18 plays a role in the pathophysiology of inflammatory arthritis [28]. On the other hand, Surdacki et al. [31], in a group of AS patients smaller than that in our study, found no differences in serum IL-18 levels between AS patients and controls. We found no differences in serum IL-18 levels between the AS, PsA, and SAPHO groups. Additionally, serum IL-18 levels were higher in male than in female SpA patients, and correlated positively with markers of inflammation, such as IL-6 in PsA and CRP in AS. Serum IL-18 correlated positively with TC in AS; this is a well-known marker of the risk of cardiovascular disease. The same was shown by Sari et al. [30]. However, there was no correlation of IL-18 with LDL-C and HDL-C in SpA patients. The results of our study suggest that active inflammation in the course of SpA is connected with an increased IL-18 level, which stimulates endothelial dysfunction and may increase the risk of atherosclerosis in this group. 

Fetuin-A plays a role in physiological and pathological mineralization [20]. Fetuin-A has an established role in extracellular matrix mineralization. In vitro, fetuin-A protects smooth muscle cells from calcification [16]. Animal studies have suggested that fetuin-A may act as an ectopic calcification inhibitor [18]. The small number of studies concerning the role of fetuin-A in the pathogenesis of endothelial dysfunction in SpA is the reason that the problem is not well recognized.

In our study, serum fetuin-A levels were lower in SpA patients than in controls. Additionally, compared to controls, SpA patients had an increased risk of decreased serum fetuin-A levels. These results are different from the results presented by Sari et al. [30], who found increased serum levels of fetuin-A in AS compared to controls. In another study of patients with AS, Tuylu et al. [32] found higher levels of fetuin-A in patients with syndesmophytes compared to AS patients without syndesmophytes and controls, but these levels were lower in patients without syndesmophytes compared to controls. There were some data showing that patients with AS had elevated levels of VEGF, which was associated with disease progression [25,26]. We found a positive correlation between fetuin-A and VEGF, which confirms the role of fetuin-A in disease progression caused by new bone formation. We can explain our results in two ways. On one hand, new bone formation—which is an intrinsic element of SpA—could reduce the concentration of serum fetuin-A in our patients. On the other hand, the decreased serum fetuin-A levels in SpA provide less of a protective effect on smooth muscle cell calcification, promoting endothelial cell dysfunction, and can increase the risk of atherosclerosis. We have also shown a negative correlation between serum fetuin-A and CRP. This confirms our hypothesis that increased disease activity is associated with greater new bone formation, and thereby results in decreased concentrations of serum fetuin-A. In our opinion, decreased fetuin-A in SpA patients could result in a decrease in its protective role in ectopic calcification, and in that way could increase the risk of endothelial dysfunction in SpA.

In our study, SpA patients had higher levels of sICAM-1 than controls, but this was not statistically significant. We found a positive correlation between serum sICAM-1 and IL-6 and ESR in SpA. Some studies have shown high sICAM-1 levels in the serum of patients with AS [2,31]. Wendling et al. [34] found a positive correlation between sICAM-1 and ESR, CRP, and IL-6 in SpA patients. Therefore, we can conclude that, in SpA serum, sICAM-1 correlates with disease activity and may have an impact on endothelial dysfunction.

ET-1 is a pro-inflammatory peptide that is mainly produced in endothelial cells, and contributes to several pathological events, including inflammation, fibrosis, and cardiac and vascular hypertrophy. The upregulation of ET-1 is regarded as an important pathogenic factor in the development of cardiovascular diseases [17]. In our study, serum ET-1 levels were lower in SpA patients than in controls. The same was found by Sari et al. [35] in AS patients. We could not find other studies that assessed ET-1 in SpA. 

We observed no influence of cigarette smoking and comorbidities such as IHD, hypertension, or diabetes on serum IL-18, fetuin-A, sICAM-1, and ET-1 levels in SpA patients. This confirms that SpA itself, not comorbidities, affected the expression of markers of endothelial dysfunction in patients with SpA in our study.

In our study, the BMI positively correlated with serum IL-18 and sICAM-1 in AS and SAPHO patients. This suggests that overweight could be connected with IL-18 and sICAM-1 production in SpA patients.

## 4. Materials and Methods

This study was approved by the Ethics Committee of the Pomeranian Medical University in Szczecin (KB-0012/106/10; 27SEP2010). 

Informed consent was obtained from all patients. We studied 191 SpA, caucasian patients: 81 had AS, 76 had PsA, and 34 had SAPHO. The controls were 30 healthy volunteers, matched to the patients according to age and sex.

The diagnosis of AS was made according to modified New York criteria, the diagnosis of PsA according to the Caspar classification criteria, and the diagnosis of SAPHO according to the Kahn criteria [10,11,12].

The following data were recorded: age, sex, disease duration, peripheral joint involvement, acute anterior uveitis (AAU), inflammatory bowel disease (IBD), psoriasis, comorbidities (hypertension, diabetes, and ischemic heart disease), cigarette smoking, and current use of medication.

Weight and height were measured to calculate the BMI (kg/m^2^). The waist and hip circumference were measured to calculate the waist/hip ratio (WHR).

Skin changes were assessed according to the Psoriasis Area and Severity Index (PASI) [36].

The patient’s pain due to the disease was assessed by a visual analogue scale (VAS).

We assessed the Bath Ankylosing Spondylitis Disease Activity Index (BASDAI). We regarded patients as active if the BASDAI score was >4 [37]. In patients with axial disease, the Ankylosing Spondylitis Disease Activity Score (ASDAS) was assessed using erythrocyte sedimentation rate (ESR) (ASDAS-ESR), calculated using the online calculator available at the Assessment of SpondyloArthritis International Society website. Disease activity score calculation in patients with peripheral arthritis was performed using a free online Disease Activity Score 28 (DAS28) calculator.

Serum was stored at −80 °C until analysis for IL-18, fetuin-A, sICAM-1, ET-1, IL-6, IL-23, VEGF, and EGF using a sensitive sandwich ELISA method: Human IL-18 Quantitative ELISA kit (MBL, Nagoya, Japan), Human Fetuin-A Immunoassay Quantikine^®^ ELISA kit, Human sICAM-1 Immunoassay Quantikine^®^ ELISA kit, Human ET-1 Immunoassay Quantikine^®^ ELISA kit, Human IL-6 Immunoassay Quantikine^®^ ELISA kit, Human IL-23 Immunoassay Quantikine^®^ ELISA kit, Human VEGF Immunoassay Quantikine^®^ ELISA kit, and Human EGF Immunoassay Quantikine^®^ ELISA kit (R&D Systems, Minneapolis, MN, USA). All analyses and calibrations were performed in duplicate and read using a BioTek PowerWaveXS spectrophotometer (Winooski, VT, USA).

Blood was taken after at least 8 h of fasting for the assessment of the erythrocyte sedimentation rate (ESR, mm/h, Westergren method), C-reactive protein (CRP, mg/dL) (turbimetric nephelometry, rate reaction), total cholesterol (TC, mmol/L), high-density lipoprotein cholesterol (HDL-C, mmol/L), low-density lipoprorotein cholesterol (LDL-C, mmol/L), and triglycerides (TG, mmol/L), measured according to standard procedures. Human leukocyte antigen B27 (HLA-B27) was determined using a BD Biosciences test (Becton, Dickinson and Company BD Biosciences, San Jose, CA, USA) based on flow cytometry and a BD FACSCanto II apparatus.

Data distributions were assessed using the Kolmogorov–Smirnov test. Data are described as mean ± standard deviation and median (Q1, Q3). We used Spearman’s rank test to calculate correlations. The *R* values of correlations were determined and corresponding *p*-values <0.05 were considered significant. The groups were compared using Student’s *t*-test, the Mann–Whitney U test, or the Kruskal–Wallis test. To assess parameters associated with serum fetuin-A, sICAM-1, IL-18, and ET-1 levels, Pearson’s χ-squared test (χ^2^), logistic regression analysis, and step-wise analysis were performed. ANOVA was performed, controlling for age and sex. The level of significance was set at *p* < 0.05. The statistical analysis was performed using STATISTICA version 8.0, StatSoft Inc., Tulsa, OK, USA.

## 5. Conclusions

In SpA patients, impaired endothelial function was found and correlated with disease activity and the lipid profile. The novelty of this work is a comprehensive assessment of a number of markers of endothelial function in combination with markers of disease activity, the lipid profile, comorbidities, and treatment in SpA.

## Figures and Tables

**Table 1:** Clinical, laboratory characteristics and treatment of the patients and controls.

Assessed Parameter	SpA Patients (*n* = 191) Mean ± SD Median (Q1, Q3)	AS Patients (*n* = 81) Mean ± SD Median (Q1, Q3)	PsA Patients (*n* = 76) Mean ± SD Median (Q1, Q3)	SAPHO Syndrome Patients (*n* = 34) Mean ± SD Median (Q1, Q3)	Controls (*n* = 30) Mean ± SD Median (Q1, Q3)
Age (years)	48.3 ± 13.1	44.7 ± 13.2	50.8 ± 12.7	51.7 ± 12.0	43.5 ± 9.4
Sex	F. 94, M. 97	F. 20, M. 61	F. 43, M. 33	F. 31, M. 3	F. 19, M. 11
Disease duration (years)	5.0 (2.0, 10.0)	10.0 (5.0, 15.0)	4.0 (2.0, 8.0)	2.0 (1.0, 5.0)	0
Peripheral arthritis, *n* (%)	97 (50.8%)	31 (38.3%)	61 (80.3%)	5 (14.7%)	0
AAU, *n* (%)	27 (14.1%)	27 (33.3%)	0 (0%)	0 (0%)	0
IBD, *n* (%)	8 (4.2%)	8 (9.9%)	0	0	0
VAS pain (mm)	51.4 ± 24.9	59.4 ± 26.8	44.9 ± 22.0	46.9 ± 20.7	0
BASDAI	4.2 ± 2.8	5.5 ± 2.6	2.8 ± 2.4	4.2 ± 2.5	0
ASDAS-ESR	2.4 (12.8, 3.2)	2.55 (2.0, 3.25)	1.8 (1.6, 2.2)	-	-
DAS28	4.24 (3.76, 4.54)	-	4.24 (3.76, 4.54)	-	-
PASI	1.0 (0.0, 3.3)	0	1.0 (0.0, 3.3)	0	0
HLA-B27 number positive/number assessed (%)	91/151 (60.3%)	75/78 (96.1%)	13/51 (25.4%)	3/22 (13.6%)	0
CRP (mg/L)	5.5 (2.1, 12.3)	6.7 (3.5, 13.6)	4.1 (1.7, 10.2)	4.3 (1.0, 12.4)	0.0
ESR (mm/h)	14.0 (6.0, 25.0)	12.0 (5.0, 26.0)	13.0 (6.0, 22.0)	19.0 (6.0, 32.0)	9.0 (2.0, 16.0)
IL-6 (pg/mL)	3.3 (1.5, 6.6)	4.06 (1.8, 7.92)	2.79 (1.47, 6.35)	2.5 (1.0, 6.61)	1.15 (0.6, 1.5)
IL-23 (pg/mL)	0.3 (0.0, 2.0)	0.3 (0.0, 2.0)	0.0 (0.0, 1.4)	0.0 (0.0, 0.3)	0.0 (0.0, 0.0)
VEGF (pg/mL)	347.4 (226.0, 562.2)	351.2 (223.2, 572.1)	343.5 (220.2, 666.4)	320.0 (240.0, 375.0)	270.0 (180.0, 445.0)
EGF (pg/mL)	104.0 (68.0, 182.0)	96.0 (68.0, 182.0)	126.0 (66.0, 196.0)	93.0 (58.0, 168.0)	81. 0 (38.0, 134.0)
Hypertension	47 (24.6%)	25 (30.9%)	13 (17.1%)	9 (26.5%)	0
Diabetes	6 (31.4%)	2 (2.5%)	1 (1.3%)	3 (8.8%)	0
IHD	19 (9.9%)	10 (12.3%)	7 (9.2%)	2 (5.9%)	0
Smoking	24 (12.6%)	12 (14.8%)	3 (3.7%)	9 (26.5%)	0
BMI	26.4 ± 4.1	26.0 ± 4.0	26.9 ± 4.2	26.8 ± 4.6	24.2 ± 3.4
WHR	0.88 ± 0.07	0.89 ± 0.08	0.88 ± 0.07	0.85 ± 0.06	-
Total cholesterol (mmol/L)	209.8 ± 40.6	200.0 ± 38.2	217.4 ± 41.5	218.1 ± 40.8	229.0 ± 40.0
HDL cholesterol (mmol/L)	59.5 ± 17.4	58.8 ± 17.7	59.4 ± 18.3	61.6 ± 15.3	62.5 ± 10.1
LDL cholesterol (mmol/L)	127.7 ± 37.0	121.1 ± 34.5	132.1 ± 40.0	135.1 ± 35.8	139.2 ± 33.8
Triglyceride (mmol/L)	129.1 ± 69.3	115.2 ± 63.3	143.5 ± 71.3	133.9 ± 75.6	135.8 ± 60.3
NSAIDs only	46 (24.1%)	25 (30.9%)	16 (21.1%)	5 (14.7%)	0 (0%)
NSADSs with sulfasalazine	90 (47.1%)	47 (58%)	31 (40.8%)	12 (35.3%)	0 (0%)
NSAIDS with methotrexate	51 (26.7%)	9 (11.1%)	25 (32.9%)	17 (50.0%)	0 (0%)
NSAIDS with methotrexate and cyclosporine	4 (2.1%)	0 (0%)	4 (5.3%)	0 (0%)	0 (0%)
Hypertensive drugs	47 (24.6%)	25 (30.9%)	13 (17.1%)	9 (26.5%)	0 (0%)

Data are presented as number (%), mean ± standard deviation, median (Q1, Q3). Abbreviations: AAU—Acute Anterior Uveitis; AS—ankylosing spondylitis; ASDAS-ESR—Ankylosing Spondylitis Disease Activity Score; BASDAI—Bath Ankylosing Spondylitis Disease Activity Index; CRP—C-reactive protein; DAS28—Disease Activity Score 28; EGF—epidermal growth factor; ESR—erythrocyte sedimentation rate; F—female; IL-6—interleukin 6; HDL: high-density lipoprotein; HLA-B27 - Human leukocyte antigen B27; IL-23—interleukin 23; IBD—inflammatory bowel disease; IHD—ischemic heart disease; LDL: low-density lipoprorotein; M—male; *n*—number of patients; NSAIDSs—nonsteroidal anti-inflammatory drugs; PASI—Psoriasis Area and Severity Index; PsA—psoriatic arthritis ; SD—standard deviation; SAPHO—Synovitis Acne Pustulosis Hyperostosis Osteitis syndrome; SpA—spondyloarthritis; VAS pain—visual analogue scale of patient’s pain; VEGF—vascular endothelial growth factor; WHR—waist/hip ratio.

**Table 2:** Serum levels of markers of endothelial function in spondyloarthritis patients groups in comparison to controls.

Assessed Parameter	SpA Patients (*n* = 191) Mean ± SD Median (Q1, Q3)	*p*-Value	* *p*-Value	AS Patients (*n* = 81) Mean ± SD Median (Q1, Q3)	*p*-Value	* *p*-Value	PsA Patients (*n* = 76) Mean ± SD Median (Q1, Q3)	*p*-Value	* *p*-Value	SAPHO Syndrome Patients (*n* = 34) Mean ± SD Median (Q1, Q3)	*p*-Value	* *p*-Value	Controls (*n* = 30) Mean ± SD Median (Q1, Q3)
IL-18 (pg/mL)	271.8 (207.3, 373.9)	0.0003	0.0116	259.7 (210.1, 372.3)	0.001	0.03	286.0 (219.6, 383.2)	0.0003	0.01	259.4 (193.3, 339.0)	0.01	0.01	198.9 (165.1, 271.5)
Fetuin-A (µg/mL)	606.0 ± 155.3	0.001	0.0059	601.0 ± 147.8	0.001	0.1	599.5 ± 152.7	0.001	0.004	632.2 ± 179.0	0.08	0.1	709.1 ± 169.3
sICAM-1 (ng/mL)	234.4 ± 61.9	0.2	0.6710	228.8 ± 57.8	0.5	0.7	237.9 ± 63.4	0.2	0.6	239.7 ± 68.7	0.2	0.7	220.0 ± 69.9
ET-1 (pg/mL)	1.24 ± 0.53	0.03	0.0213	1.13 ± 0.38	0.0005	0.0003	1.25 ± 0.58	0.08	0.02	1.49 ± 0,64	0.87	0.82	1.47 ± 0.57

Data are presented as mean ± standard deviation (SD), median (Q1, Q3). The Student *t*-test was used for parameters with a normal distribution, Mann–Whitney test was used for parameters with a lack of normal distribution. * age–sex adjusted *p*-value for the defining groups of patients in ANOVA. AS—ankylosing spondylitis; ET-1—endothelin-1; IL-18—interleukin 18; PsA—psoriatic arthritis; SAPHO—Synovitis Acne Pustulosis Hyperostosis Osteitis syndrome; sICAM-1—soluble intercellular adhesion molecule-1; SpA—spondyloarthritis.

**Table 3:** A logistic regression model of the odds ratio of the increased serum levels of interleukin-18, decreased serum levels of fetuin-A, increased serum levels of soluble intercellular adhesion molecule-1, and decreased serum levels of endothelin-1 in the spondyloarthritis group of patients compared to controls.

Covariates	IL-18 ≥ 227.45 pg/mL		Fetuin-A ≤ 608.5 µg/mL		sICAM-1 ≥ 172.95 ng/mL		ET-1 ≤ 1.081 pg/mL	
	OR (95% CI)	*p*	OR (95% CI)	*p*	OR (95% CI)	*p*	OR (95% CI)	*p*
SpA	3.22 (1.37–7.55)	0.007	4.14 (1.59–10.81)	0.004	2.7 (1.07–6.78)	0.03	6.73 (2.13–21.27)	0.001
AS	2.31 (0.84–6.32)	0.1	2.63 (0.87–7.92)	0.09	3.75 (1.03–13.74)	0.04	10.24 (2.89–36.23)	<0.0001
PsA	4.07 (1.53–10.82)	0.005	5.5 (1.91–15.91)	0.002	3.08 (1.03–9.23)	0.04	7.01 (2.02–24.34)	0.002
SAPHO	3.08 (0.98–9.65)	0.05	3.89 (1.12–13.56)	0.03	1.87 (0.55–6.41)	0.3	3.07 (0.69–13.73)	0.142

AS—ankylosing spondylitis; ET-1—endothelin-1; IL-18—interleukin 18; OR—odds ratio; PsA—psoriatic arthritis; sICAM-1—soluble intercellular adhesion molecule-1; SAPHO—Synovitis Acne Pustulosis Hyperostosis Osteitis syndrome; SpA—spondyloarthritis.
